# Preparation and Electrochemical Characterization of a Carbon Ceramic Electrode Modified with Ferrocenecarboxylic Acid

**DOI:** 10.3390/s110201361

**Published:** 2011-01-25

**Authors:** Tatiane Skeika, Cristiane R. Zuconelli, Sergio T. Fujiwara, Christiana A. Pessoa

**Affiliations:** 1 Department of Chemistry, Universidade Estadual Ponta Grossa, Avenida Carlos Cavalcanti 4748, Uvaranas, Ponta Grossa, Brazil; E-Mails: tatiuepg@hotmail.com (T.S.); criszuconelli@hotmail.com (C.R.Z.); 2 Department of Chemistry, CEDETEG—Universidade Estadual do Centro-Oeste, Rua Presidente Zacarias 875, Guarapuava, Brazil; E-Mail: sergiofujiwara@yahoo.com.br (S.T.F.)

**Keywords:** carbon ceramic electrode, ferrocene carboxylic acid, dopamine

## Abstract

The present paper describes the characterization of a carbon ceramic electrode modified with ferrocenecarboxylic acid (designated as CCE/Fc) by electrochemical techniques and its detection ability for dopamine. From cyclic voltammetric experiments, it was observed that the CCE/Fc presented a redox pair at *E*_pa_ = 405 mV and *E*_pc_ = 335 mV (Δ*E* = 70 mV), related to the ferrocene/ferrocenium process. Studies showed a considerably increase in the redox currents at the same oxidation potential of ferrocene (*E*_pa_ = 414 mV *vs.* Ag/AgCl) in the presence of dopamine (DA), differently from those observed when using only the unmodified CCE, in which the anodic peak increase was considerably lower. From SWV experiments, it was observed that the AA (ascorbic acid) oxidation at CCE/Fc occurred in a different potential than the DA oxidation (with a peak separation of approximately 200 mV). Moreover, CCE/Fc did not respond to different AA concentrations, indicating that it is possible to determine DA without the AA interference with this electrode.

## 1. Introduction

The construction of electrodes using porous materials such as carbon ceramic electrodes (CCE), has seen a great development since the last decade [1–5]. The CCEs are a class of materials with high electrical conductivity which were firstly described by Lev *et al.* [6]. These electrodes are basically constructed by doping a silica matrix obtained by the sol-gel method with powdered carbon such as graphite, or other carbon materials (carbon nanotubes and glassy carbon) [7,8]. The advantage of using these materials compared to other carbon-based electrodes is the combination of the sol-gel process properties (such as high surface area) and conductivity of the carbon materials, thus enabling one to obtain a renewable surface electrode similar to a carbon paste electrode, but more robust and with higher stability [9–11].

An alternative to increase the application of carbon ceramic electrodes as electrochemical sensors is the modification of these materials using electron mediator species, such as organic or inorganic complexes and enzymes [12–14]. These mediators can provide new interesting features to these electrodes, such as the shift of peak potential of the analyte species to less positive potentials, thus increasing the sensitivity of the electrode [15–17]. Recently, a CCE modified with SnO_2_ and cobalt phthalocyanine was described. The obtained material resulted in a homogeneous material with good dispersion, which was applied to oxalic acid determination at 0.84 V (SCE) [18]. In this context, Salimi and Abdi constructed an amperometric sensor for hydrazine and hydroxylamine detection based on CCE modification with powdered nickel and subsequent deposition film of nickel hexacyanoferrate. The analytical parameters obtained for the modified CCE showed better results than those obtained for the unmodified CCE, justifying the advantage of electrode modification for determination of species of environmental interest [19]. Another interesting application for the carbon ceramic composite is related to the construction of biosensors. The detection of glucose was proposed by Tian *et al.*, who developed a sensor consisting of polypyrrole (ppy) electrochemical deposition in the presence of glucose oxidase on the surface of a CCE with HRP incorporated during the sol-gel process. This biosensor has shown considerable sensibility to the analyte, with a high stability within the period of three weeks [20].

Ferrocene-derived compounds are examples of electrons mediators highly used in electrochemical systems and also in enzymatic reactions [21,22]. ascorbate determination [23]. dopamine [24]. pH and oxygen sensing [25]. Fernandez and Carrero reported a glassy carbon chemically modified using surfactant/clay films, containing ferrocenedicarboxylic acid. The results showed that low concentrations of ascorbic acid and uric acid are easily oxidized by the proposed electrode [26].

This paper reports the development of a CCE modified with ferrocenecarboxylic acid (CCE/Fc). The electrode was characterized by voltammetric techniques and applied as an electrochemical sensor for dopamine, which is an important neurotransmitter and is related to several diseases such as schizophrenia and parkinsonism [27,28]. Therefore, the development of methods for the quantification of dopamine in blood and biological fluids is the subject of intense current investigation [29–31]. The importance of using a carboxylated ferrocene derivative as a mediator for DA detection is related to the fact that the carboxylic groups depending of the solution pH could facilitate the interaction between this analyte and the electrode surface. Furthermore, the combination of such mediator with carbon ceramic composite electrodes can provide interesting properties and therefore increase the number of technological applications of these materials, especially in the field of electrochemical sensors.

## 2. Experimental Section

### 2.1. Preparation of Carbon Ceramic Electrode

The CCE/Fc was prepared according to the procedure previously described by Lev and co-workers [6]. The hydrolyzed mixture (where groups of the alkoxide precursor are converted to silanol groups) consisted of 1.5 mL of methanol (Aldrich), 1.2 mL of precursor, MTMOS (methyltrimethoxysilane) (Aldrich) and 15 μL of 12 mol L^−1^ HCl (Synth). To this mixture, three different quantities of ferrocenocarboxilic acid (2.5, 5.0 and 10 mg) and 1.8 g of graphite (Fluka) were added. The non-modified CCE was prepared by using the same experimental procedure used for CCE/Fc, without the ferrocenocarboxylic acid mixing step. The resulting sol was introduced into a glass tube (exposed area of approximately 0.2 cm^2^). A nickel-chromium (Ni-Cr) wire was inserted for electrical contact. The electrodes were subjected to the drying process at room temperature during one week.

### 2.2. Electrochemical Studies

The electrochemical measurements were carried out in a PalmSens potentiostat, connected to a computer for data acquisition, with the conventional three electrode system: platinum counter electrode, reference electrode (Ag/AgCl), and working electrodes (CCE and CCE/Fc).

The electrochemical characterization was performed by cyclic voltammetry, in supporting electrolyte (NaCl 0.5 mol L^−1^, pH = 6.0), using a 10 mL capacity electrochemical cell varying the scan rate (in the range of 10–100 mVs^−1^). The electrode response in presence of dopamine (DA) was also studied by cyclic voltammetry and square wave voltammetry in 0.1 mol L^−1^ phosphate buffer (pH = 7.0), which was used as supporting electrolyte. The dopamine solution was daily prepared in a concentration of 10 mmol L^−1^. Square wave voltammetry experiments were also evaluated in presence of DA and AA in different concentrations in the range from 1.0 to 2.5 μmol L^−1^. Studies in different pH (pH = 1.0 to 7.0) were evaluated using NaCl 0.5 mol L^−1^ solution. The adjustment of pH was carried out by NaOH or HCl addition.

### 2.3. Scanning Electron Microscopy and EDS Mapping

Morphological characterization of CCE/Fc was carried out by the techniques of scanning electron microscopy and energy dispersive spectroscopy (SEM and EDS), using a Shimadzu SSX-550 instrument. The samples were fixed on the sample holder using double-faced conductive graphite tape, without the need of a pretreatment, since the carbon ceramic has conductive properties. The CCE/Fc micrographs were obtained with a magnification of 500 X and 1,500 X in the range of 20 and 100 μm.

## 3. Results and Discussion

### 3.1. Scanning Electron Microscopy and EDS Mapping

**Figure 1.**
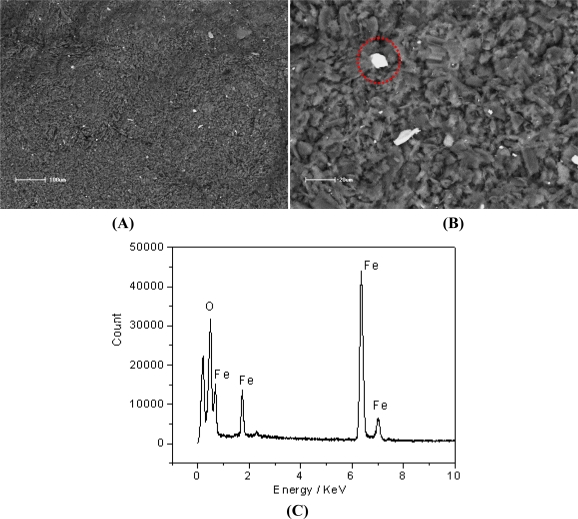
EDS images for Fe mapping **(A)** 500 X and **(B)** 1,500 X and the corresponding **(C)** EDS analysis.

Figure 1 shows the energy dispersive scanning images of Fe in CCE/Fc within magnifications of 500 X and 1,500 X (Figure 1A,B) and the corresponding EDS analysis (Figure 1C). From, the EDS images, it can be observed that the components are homogeneously dispersed in the sample but with some agglomerations of segregated ferrocenecarboxylic molecules. In order to confirm that the bright regions of the obtained EDS images were related to the ferrocenecarboxylic particles, a quantitative microanalysis of the highlighted region (circle with a dotted line in Figure 1B) was realized. The EDS analysis indicated a 72.38% w/w of Fe in the sample (Figure 1C).

### 3.2. Electrochemical Studies of the CCE/Fc

#### 3.2.1. Electrochemical Characterization of the CCE/Fc

**Figure 2.**
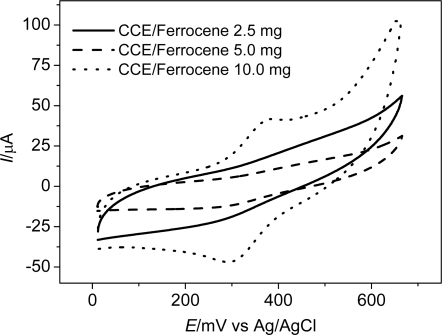
Cyclic voltammograms of the CCE/Fc with different amounts of ferrocene: 0.0025 g; 0.005 g and 0.010 g (ν = 50 mV s^−1^ and [NaCl] = 0.5 mol L^−1^).

The carbon ceramic electrode was modified with three different amounts of ferrocene (0.0025 g; 0.005 g and 0.010 g), and designated as CCE/Fc. From the cyclic voltammetric studies, it was verified that only the CCE modified with the highest quantity of ferrocene presented redox peaks, with *E*_pa_ = 405 mV and *E*_pc_ = 335 mV (Δ*E* = 70 mV), as shown in Figure 2. These peaks are probably related to the ferrocene/ferrocenium redox process, since the non modified CCE did not present any redox peaks [26].

**Figura 3.**
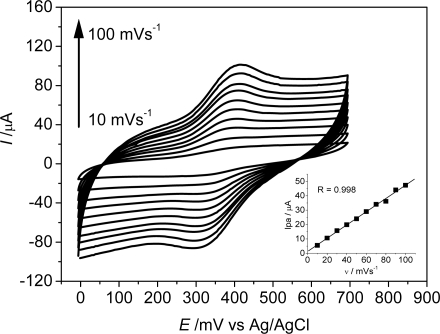
Cyclic voltammograms of the CCE/Fc at different scan rates (10 to 100 mV s^−1^). Inserted figure: Relationship between *I*_pa_ *versus* ν. Supporting electrolyte: [NaCl] = 0.5 mol L^−1^.

Voltammetric studies at different scan rates resulted in a linear relation between the anodic peak current values and the scan rate (Figure 3), which is a characteristic behavior for confined species in the bulk of the electrode, indicating that the electroactive species are strongly entrapped in the electrode surface [32].

**Figure 4.**
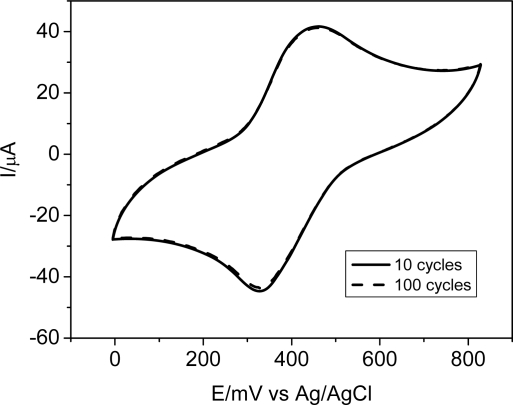
CCE/Fc stability after repeated 100 cycles. Supporting electrolyte: [NaCl] = 0.5 mol L^−1^.

In order to verify the stability of the modified electrode, consecutive voltammograms were obtained in the presence only of the electrolyte solution at a fixed scan rate (ν = 50 mV s^−1^). The voltammetric profile was analyzed as a function of the anodic current peak values (Figure 4). After repeated cycles (approximately 100), the current response of the bulk-modified carbon ceramic electrode did not decrease substantially (with a 0.22% variation, shown in Figure 4), indicating that the modified CCE is quite stable and can be used as an electrochemical sensor. The reproducibility of the electrode response was also studied as a function of the days (in the electrolyte solution) for one month. In this case, the *I*_pa_ variation was of 1.42 % and for Epa 0.18%, showing that the electrode holds its efficiency within this period of time.

The pH influence in the electrochemical response of the CCE/Fc was also verified. It was observed that with the increase of pH from 2 to 7, the peak potentials negatively shift from 520 to approximately 420 mV (figure not shown). However in this pH range, the peak currents remained practically constant. This can be explained considering that the ferrocenecarboxylic acid redox reaction involves protons [33], with a p*K*_a_ = 4.2. When the pH decreases, it becomes more difficult to oxidize the ferrocenecarboxylic and peak potentials are shifted to more positive values.

#### 3.2.2. Electrochemical Studies of the CCE/Fc in Presence of Dopamine

In order to verify the possibility of using this modified electrode as an electrochemical sensor, studies in presence of dopamine (DA) were carried out. The voltammograms obtained in the absence of DA, presented only the redox peaks related to the redox process of ferrocenecarboxylic acid. However, after addition of DA in the electrolyte solution, a considerable increase in the redox currents was observed at approximately the same potential of the mediator species (*E*_pa_ = 414 mV).

**Figure 5.**
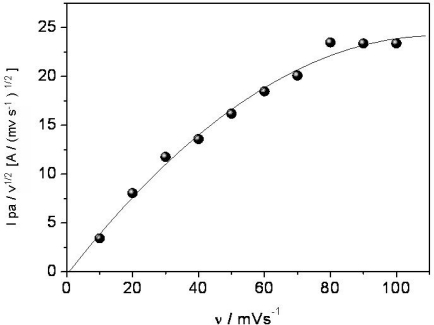
Plot of *I*_pa_/ν^1/2^ against scan rate for CCE/ferrocene in presence of [DA] = 1.0 × 10^−3^ mol L^−1^. Supporting electrolyte: phosphate buffer (0.1 mol L^−1^) pH = 7.0.

The electrocatalytic response was confirmed by performing a blank test, in the absence of the ferrocenecarboxylic acid, *i.e.*, using CCE (instead of modified CCE) as electrode material. In this case, the increase in the anodic peak was considerably lower, even in the presence of 10 × 10^−3^ mol L^−1^ of DA and slightly dislocated to higher positive potential (*E*_pa_ = 448 V). The plot of peak current against the square root of the scan rate in the range of 10–100 mV s^−1^ showed a linear relation (with *r* = 0.996), indicating a diffusion controlled process, what is expected for catalytic systems and it is advantageous for quantitative measurements. Moreover, in order to confirm that DA oxidation occurred by an electrocatalytical process when the CCE/Fc was employed, the dependence of the parameter *I*_pa_/ν^1/2^ on scan rate was plotted, as shown in Figure 5. According to Nicholson and Shain [34] a non-linear relationship of the plot *I*_pa_/ν^1/2^ *vs.* ν exhibit the characteristic shape of a typical EC (electrochemical-chemical) catalytic process, as observed for the CCE modified with ferrocenecarboxylic acid. These results are similar to those obtained for other electrocatalytical systems for DA oxidation [27].

**Figure 6.**
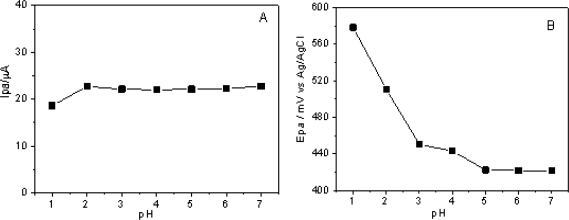
Effect of pH of the supporting electrolyte NaCl 0.5 mol L^−1^ on the *I*_pa_ values **(A)**, and *E*_pa_ values **(B)** of the CCE/Fc in presence of [DA] = 1.0 mmol L^−1^.

The redox process of DA also involves protons, hence the pH of the electrolyte is an important parameter which influences its oxidation [35]. From Figure 6A, it can be observed that the current is not significantly influenced by the pH differences, however the peak potential is shifted considerably to high positive potentials as the pH decreases (Figure 6B). The p*K*_a_ for DA molecule is 8.9, which means that at the studied pH range, the reduced form of dopamine (cationic form) is predominant [36]. Therefore, as the pH decreases, the DA oxidation reaction becomes more difficult, which causes the shift of Epa to more positive potentials values, as can be observed in Figure 6B.

The pH chosen for further measurements was 7.0, since that in this condition *E*_pa_ is not dislocated much to higher positive potentials and it is close to the physiological pH, what favors the application of the modified electrode in living systems.

**Figure 7.**
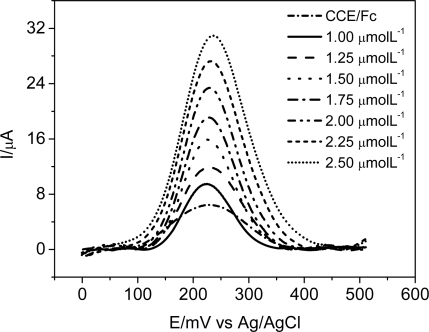
Square wave voltammograms of CCE/Fc at different DA concentrations in the range of 1.0 a 2.5 μmol L^−1^ in 0.1 mol L^−1^ phosphate buffer at pH 7.0. SWV Parameters: *f* = 60 s^−1^, a = 60 mV e Δ*E* = 2.0 mV.

In order to obtain the analytical parameters for the modified CCE, square wave voltammetry (SWV) experiments were carried out due to its higher sensibility when compared to the cyclic voltammetry technique, at several DA concentrations in 0.1 molL^−1^ phosphate buffer (pH 7.0). Firstly, the SWV parameters were optimized to obtain the best conditions for DA determination. The effect of frequency changes on the peak current was studied between the range of 10–100 s^−1^ and the results showed that the frequencies up to 60 s^−1^ cause an increase in peak current. However, at higher frequencies the currents decrease considerably. Therefore, f = 60 s^−1^ was chosen as optimum frequency value. The other effect studied was pulse amplitude in the range of 10–100 mV (with 10 mV intervals). The results obtained showed that, increasing the pulse heights up to 60 mV, an increase in peak current will be caused. Pulse heights higher than 60 mV causes broadening of the CCE/Fc voltammogram and the decreasing of the analyte peak current intensity. The effect of scan increment, which determines the amount of potential changes between two data points in the experiment, was also investigated. Scan increment values in the range of 1.0–10 mV (with 1 mV intervals) were applied to the electrodes and the corresponding voltammograms were recorded. The resulting peak currents showed that, by increasing the scan increment up to 2.0 mV values, the voltammograms’ peak current will also increase steadily. Therefore, the best conditions encountered were: *f* = 60 s^−1^ a = 60 mV e Δ*E* = 2.0 mV. Besides the higher sensitivity of the technique SWV, it was observed a shift of oxidation peak potentials to more negative regions (approximately *E*_p_ = 237 mV). This shift is described by Souza *et al.* as a result of scan rate. An explanation for this behavior is that, when high scan rates are applied, the influence of the increment (Δ*E*) is increased and can cause displacement in values of peak potentials [37]. Using these optimized conditions, the proposed sensor has shown a linear response range from 1.0 to 2.5 μmol L^−1^ (Figure 7), which can be expressed according to the following equation: *I*_p_ (μA) = −6.31 + 1.47 × 10^7^ [DA/mol L^−1^] (R = 0.997). A detection limit of 0.45 μmol L^−1^ was determined using a 3σ/slope ratio, where σ is the standard deviation calculated from the ten background current values (blank measurements), determined according to IUPAC recommendations [38].

**Figure 8.**
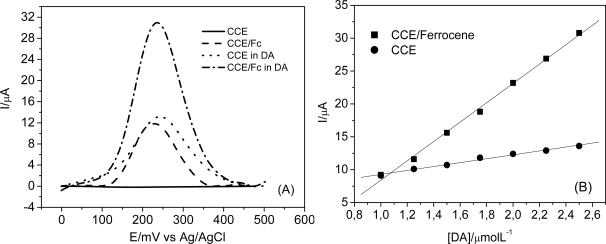
**(A)** SWV of CCE/Fc and CCE in absence and presence [DA] = 2.5 μmol L^−1^ in 0.1 mol L^−1^ phosphate buffer at pH 7.0. **(B)** Analytical curves obtained with CCE/Fc and CCE at different DA concentrations in the range of 1.0 a 2.5 μmol L^−1^ in 0.1 mol L^−1^ phosphate buffer at pH 7.0. SWV Parameters: *f* = 60 s^−1^, a = 60 mV e Δ*E* = 2.0 mV.

When compared to the non-modified CCE, the CCE/Fc has showed a lower detection limit and higher sensitivity for DA determination, as can be seen in Figure 8. This behavior can probably be related to the affinity between the electrocatalyst (ferrocenecarboxylic acid) and the analyte (DA), which consequently enhances the efficiency of the electron transfer processes. Ferrocene derived compounds have been used as electron mediators in modification of carbon paste electrodes [39], glassy carbon [40] and also in the preparation of films on gold surfaces for determination of DA [41].

**Figure 9.**
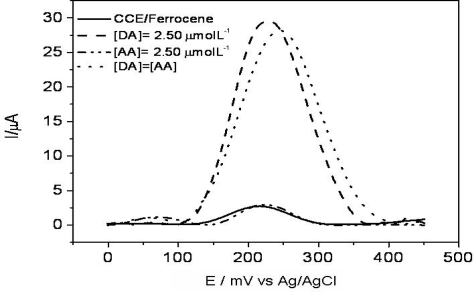
SWV of CCE/Fc in absence and presence of AA and DA, both in a concentration of 2.5 μmol L^−1^ in 0.1 mol L^−1^ phosphate buffer at pH 7.0. [AA] = [DA] simultaneous additions in same concentrations. SWV Parameters: *f* = 60 s^−1^, a = 60 mV e Δ*E* = 2.0 mV.

The influence of ascorbic acid (AA) in the electrochemical oxidation of DA was evaluated using the SWV technique. According to Figure 9, using this technique the CCE/Fc presented only one peak which appeared at 230 mV. After DA addition, a considerable current peak increase is observed at the same potential. In contrast, in the presence of AA, a peak appeared at 50 mV, probably due to the AA oxidation at the CCE/Fc. Therefore, different from described in the literature, the current peak did not increase with AA concentration until 1.0 mmol L^−1^ [42]. This behavior can be explained considering that as the p*K*_a_ of ferrocenecarboxylic is 4.2, in the electrolyte solution at pH 7.0, the anionic form of AA is predominant and it is responsible for the electrochemical response. At these conditions the CCE/Fc probably repels the AA species, hindering their approximation to the electrode surface, and causing a decrease of the electrode sensitivity for these species compared to the DA molecules (which is in the cationic form at pH 7.0).

When DA and AA were added simultaneously in the electrochemical cell, two peaks were observed, one at 50 mV and another at 250 mV related to AA and DA, respectively. These results indicated that, using the CCE/Fc it is possible to detect DA without AA interference, since the AA oxidizes at a different potential from DA (with a peak separation of approximately 200 mV) and the AA potential does not increase linearly with concentration.

The analytical parameters obtained are comparable with results reported in the literature for determination of DA and AA at the surface different composite electrodes (Table 1), although its linear response range is considerably narrow and should be improved.

## 4. Conclusions

In this paper, we have presented a carbon ceramic electrode prepared by the sol–gel technique and modified with ferrocenecarboxylic acid. SEM images coupled to surface element mapping (EDS) indicated that the components are homogeneously dispersed in the sample but with some agglomeration of ferrocene species. The electrochemical results demonstrated that the CCE/Fc is capable of determining dopamine with good sensitivity and selectivity compared to other similar carbon-based electrodes. Moreover, square wave voltammetric experiments realized in the presence of both AA and DA showed two peaks one at 50 mV and another at 250 mV (related to AA and DA, respectively). These results indicated that using the CCE/Fc it is possible to detect DA without AA interference, since the AA oxidizes at a different potential of DA (with a peak separation of approximately 200 V) and the AA potential does not increase linearly with concentration. CCE/Fc is a robust and stable material which is likely to be used on the construction of electrochemical sensors, also coupled with enzymes, which can be easily incorporated in the matrix surface.

## Figures and Tables

**Table 1.** Analytical parameters for carbon composite electrodes for detection of DA and AA.

**Electrode**	**Method**	**Analytes**	**Linear range (μmol L^−1^)**	**Limit of detection (μmol L^−1^)**	**Sensitivity (μA/μmol L^−1^)**	**Ref.**
Carbon ceramic composite	SWV	DA	0.5–20	0.1	0.75	30
		AA		0.1	0.754	
Methylene blue- zirconia-silica mixed oxide	DPV	DA in presence of AA	40–160	4.0	0.47	18
MWCCE *	Cyclic voltammetric	DA	4–1,000	1.5	0.13	43
Ferrocene-Pd-ormosil	Amperometry	DA	1,000–8,000	50	0.13	44
**CCE/Ferrocene**	SWV	DA	1–2.5	0.45	14.7	This work
		AA		—	—	

* sol-gel carbon ceramic electrode (CCE) prepared by microwave (MW) irradiation
